# Unravelling the Importance of Diazotrophy in Corals – Combined Assessment of Nitrogen Assimilation, Diazotrophic Community and Natural Stable Isotope Signatures

**DOI:** 10.3389/fmicb.2021.631244

**Published:** 2021-06-24

**Authors:** Vanessa N. Bednarz, Jeroen A. J. M. van de Water, Renaud Grover, Jean-François Maguer, Maoz Fine, Christine Ferrier-Pagès

**Affiliations:** ^1^Marine Department, Centre Scientifique de Monaco, Monaco City, Monaco; ^2^Laboratoire des Sciences de l’Environnement Marin (LEMAR), UMR 6539 UBO/CNRS/IRD/IFREMER, Institut Universitaire Européen de la Mer, Plouzané, France; ^3^Mina and Everard Goodman Faculty of Life Sciences, Bar-Ilan University, Ramat Gan, Israel; ^4^The Interuniversity Institute for Marine Sciences, Eilat, Israel

**Keywords:** nitrogen fixation, stable isotopes, corals, mesophotic coral reef, *nifH* amplicon sequencing, 16S rRNA gene, diazotroph, microbiome

## Abstract

There is an increasing interest in understanding the structure and function of the microbiota associated with marine and terrestrial organisms, because it can play a major role in host nutrition and resistance to environmental stress. Reef-building corals live in association with diazotrophs, which are microbes able to fix dinitrogen. Corals are known to assimilate diazotrophically-derived nitrogen (DDN), but it is still not clear whether this nitrogen source is derived from coral-associated diazotrophs and whether it substantially contributes to the coral’s nitrogen budget. In this study, we aimed to provide a better understanding of the importance of DDN for corals using a holistic approach by simultaneously assessing DDN assimilation rates (using ^15^N_2_ tracer technique), the diazotrophic bacterial community (using *nifH* gene amplicon sequencing) and the natural δ^15^N signature in *Stylophora pistillata* corals from the Northern Red Sea along a depth gradient in winter and summer. Overall, our results show a discrepancy between the three parameters. DDN was assimilated by the coral holobiont during winter only, with an increased assimilation with depth. Assimilation rates were, however, not linked to the presence of coral-associated diazotrophs, suggesting that the presence of *nifH* genes does not necessarily imply functionality. It also suggests that DDN assimilation was independent from coral-associated diazotrophs and may instead result from nitrogen derived from planktonic diazotrophs. In addition, the δ^15^N signature presented negative values in almost all coral samples in both seasons, suggesting that nitrogen sources other than DDN contribute to the nitrogen budget of corals from this region. This study yields novel insight into the origin and importance of diazotrophy for scleractinian corals from the Northern Red Sea using multiple proxies.

## Introduction

Corals live in symbiosis with a variety of microorganisms, including unicellular algae, bacteria, fungi, and archea, collectively termed the “coral holobiont” (Knowlton and Rohwer, 2003; Wegley et al., 2007). Coral-associated microbes maintain important functions for the coral holobiont, including nutrient acquisition, and antimicrobial defense (Bourne et al., 2016). In many shallow and mesophotic coral species, unicellular algae (Symbiodiniaceae) are often important contributors to the carbon requirements of corals through photoautotrophic carbon input. In addition, they are essential for the recycling of nutrients such as nitrogen within the coral symbiosis, since nitrogen availability is often limited in oligotrophic coral reef waters (de Goeij et al., 2013). Besides internal recycling and an efficient uptake of nitrogenous compounds from the surrounding seawater, biological dinitrogen (N_2_) fixation, the conversion of atmospheric N_2_ into bioavailable nitrogen (i.e., ammonium), represents an additional source of new nitrogen for corals (Cardini et al., 2014; Benavides et al., 2017). It has been demonstrated that corals obtain diazotrophically-derived nitrogen (DDN) from planktonic N_2_ fixing bacteria (i.e., diazotrophs) (Benavides et al., 2016, 2017; Meunier et al., 2019). In addition, diazotrophs have been identified in several coral species as part of their coral microbiome (e.g., Lema et al., 2012; Lesser et al., 2018, 2019; Bednarz et al., 2019), but the nutritional importance of these associated bacteria in providing DDN to the coral holobiont is still unclear.

Several studies assessed indirectly the gross N_2_ fixation associated with corals (i.e., acetylene reduction assay) or directly the net DDN assimilation by corals (i.e., ^15^N_2_ tracer technique) to demonstrate that coral-associated diazotrophs are actively fixing N_2_ (Table 1). However, to what extent DDN contributes to the coral’s nitrogen budget is still unclear, because it is difficult to distinguish between the nitrogen assimilated by the diazotrophs located within the coral tissue/mucus and the DDN assimilated in the coral tissues. In addition, the determination of both gross N_2_ fixation and DDN assimilation requires short term *in vitro* incubation experiments, which only represent a snapshot of the actual importance of DDN for coral metabolism. Instead, the natural δ^15^N signature of coral tissue can give insights into the main nitrogen sources used by corals on a much larger time scale. Although the δ^15^N technique cannot be used to directly assess the amount of DDN assimilated by corals, a δ^15^N signature in the coral tissue slightly higher than the δ^15^N value of atmospheric N_2_ (0‰), or of the diazotroph community (2–3‰) may indicate that N_2_ fixation is the main nitrogen source (Alamaru et al., 2009; Hadas et al., 2009). Both, δ^15^N and δ^13^C isotope ratios are useful indicators to trace food sources and metabolic pathways in corals by distinguishing carbon and nitrogen fixation derived from Symbiodiniaceae activity or heterotrophic feeding. Consequently, to evaluate the relative importance of DDN for the coral’s nitrogen budget, the actual DDN assimilation rates, and the natural δ^15^N-δ^13^C signature need to be assessed and compared simultaneously.

**TABLE 1:** Overview of studies that quantified DDN assimilation in scleractinian corals using the ^15^N_2_ tracer technique.

**Coral**	**Location**	**ng N cm**^–^**^2^ h**^–^**^1^**	**Diazotroph origin**	**References**
*Stylophora pistillata*	Red Sea	0	Coral-associated	Grover et al. (2014)
*Stylophora pistillata*	New Caledonia	200–500	Coral-associated	Benavides et al. (2016)
*Stylophora pistillata*	Red Sea	1–10	Coral-associated	Bednarz et al. (2017)
*Stylophora pistillata*	GBR	6–16	Coral-associated	Lesser et al. (2018)
*Oculina patagonica*	Mediterranean	4–12	Coral-associated	Bednarz et al. (2019)
*Montipora capitata*	Hawaii	8–17	Coral-associated	Lesser et al. (2019)
*Montastraea cavernosa*	Caribbean	20–70	Coral-associated	Lesser et al. (2019)
*Stylophora pistillata*	New Caledonia	600–900	Planktonic	Benavides et al. (2016)
*Stylophora pistillata*	New Caledonia	0–1000	Planktonic	Meunier et al. (2019)

*DDN was either derived from coral-associated or planktonic diazotrophs.*

The availability of N_2_ fixation products for corals varies depending on the prevailing environmental conditions that influence the activity and community composition of diazotrophs. In the Northern Red Sea, the gross N_2_ fixation associated with coral holobionts increases when reactive nitrogen (i.e., ammonium, nitrate, and dissolved organic nitrogen) availability in seawater is low, for example during the summer (Bednarz et al., 2015; Cardini et al., 2015). Such differences in N_2_ fixation in coral holobionts may also be linked to changes in the coral-associated diazotrophic community. The composition of these communities is known to vary between coral species, environmental conditions and geographical location (Lema et al., 2014b; Zhang et al., 2016; Lesser et al., 2018; Liang et al., 2020). In particular, seasonal shifts of ammonium and nitrate in the surrounding seawater have been suggested to drive seasonal changes in diazotrophic communities associated with corals (Zhang et al., 2016). This suggests that diazotrophs may indeed play an important role in the nitrogen provision to the coral holobiont when reactive nitrogen availability is low. Therefore, studying seasonal dynamics of both coral-associated diazotrophs and DDN assimilation rates in corals is important to better evaluate the relative importance of diazotrophs for the nitrogen budget of coral holobionts under different environmental conditions.

In this study, we aimed to provide a better understanding of the importance of DDN for corals using a holistic approach. We simultaneously assessed DDN assimilation rates, the natural δ^15^N signature (as a proxy for nitrogen source) and the diazotrophic bacterial community in the holobiont of the reef-building coral *Stylophora pistillata* from the Northern Red Sea along a depth gradient in winter and summer (characterized by seasonal differences in reactive nitrogen availability). As nitrogen is one of the key nutrients explaining the success of corals in oligotrophic environments, as well as their resistance to environmental stressors, a better knowledge of the sources of nitrogen available for coral growth is needed in an era of rapid climate change and stress imposed on coral reefs.

## Materials and Methods

### Coral Collection and Maintenance

The study was conducted at the Interuniversity Institute for Marine Science (IUI), Northern Gulf of Aqaba, Israel, in winter (November 2016), and summer (August 2017). In each season, large fragments were collected from six different colonies of *Stylophora pistillata* at 5, 25, and 50 m water depth (*n* = 6 per depth) using SCUBA. *S. pistillata* was chosen as it is a common species in the Gulf of Aqaba, a depth generalist, and an established coral model for N_2_ fixation studies (Benavides et al., 2016; Bednarz et al., 2017, 2018; Lesser et al., 2018; Meunier et al., 2019). Each fragment was cut into 3 smaller fragments (10 to 30 cm^2^ surface area) for (1) ^15^N_2_ incubation experiments to quantify DDN assimilation by the corals, (2) the natural δ^15^N signature, and (3) the determination of the coral-associated diazotroph and overall prokaryotic communities. Fragments for the microbial community analyses were rinsed with 0.22 μm filtered seawater (Whatman Membrane Filter) to remove exogenous bacterial contaminants and stored in RNA*later* (Thermo Fisher Scientific) at 4°C until further processing. The remaining coral fragments were maintained in 9 outdoor flow-through holding tanks in the Red Sea Simulator (Bellworthy and Fine, 2018) until the ^15^N_2_ incubation experiments (described below) on the following day. The tanks were exposed to the natural light cycle and light conditions (daily maximum) in the tanks were adjusted according to the *in situ* levels (μmol photons m^–2^ s^–1^) at 5 m (summer: 950 ± 20; winter: 750 ± 20), 25 m (summer: 330 ± 10; winter: 250 ± 10), and 50 m (summer: 80 ± 2; winter: 60 ± 2) by hanging layers of black shade cloth above the tanks (3 tanks per light condition). Seawater in the tanks was directly pumped from the reef ensuring *in situ* water temperature (summer: 26.9 ± 0.1°C; winter: 24.8 ± 0.1°C), dissolved inorganic nitrogen (DIN; summer: 112 ± 44 nM DIN; winter: 280 ± 183 nM DIN), phosphate (summer: 47 ± 9 nM phosphate; winter: 43 ± 4 nM phosphate), and chlorophyll *a* (summer: 127 ± 21 ng L^–1^ Chl *a*; winter: 390 ± 10 ng L^––1^ Chl *a*) concentrations in each season. Environmental data were provided by the Israel National Monitoring Program at the Gulf of Aqaba^1^ and represent average values (mean ± SD) of seasurface samples collected during November 2016 and August 2017 at three coastal sampling stations (Taba, Japanese Gardens, Water Control Station) close to our study site. Additional data from an open-water monitoring station (∼3 km away from the study site) were inspected and confirmed that water temperature and nutrient concentrations within each season were stable within the 5 to 50 m water depth range.

### ^15^N_2_ Incubation Experiments

We used the ^15^N_2_-enriched seawater addition method to quantify DDN assimilation by the corals (Mohr et al., 2010). For this, ^15^N_2_-enriched seawater was produced prior to the incubation experiment by injecting 10 ml of ^15^N_2_ gas (98% Eurisotop) into 500 ml degassed 0.2 μm filtered seawater followed by vigorous shaking for at least 12 h. This procedure ensures 90 to 100% ^15^N_2_ equilibration (Mohr et al., 2010). In addition, 6 L of seawater were collected from each water depth, respectively, using Niskin bottles. For the incubations, 500 ml beakers were filled with 450 ml of the depth-corresponding unfiltered seawater and 1 coral fragment was placed into each bottle (*n* = 6). Thereafter, 50 ml ^15^N_2_ enriched seawater was added to each beaker and beakers were immediately closed gas-tight. The remaining coral fragments (*n* = 6 per depth) were incubated in 500 ml seawater (without addition of ^15^N_2_ enriched seawater) and served as controls for the ^15^N_2_ assimilation experiment as well as to determine the natural isotopic δ^15^N and δ^13^C signatures of the corals. All bottles were incubated under constant stirring for a full dark-light cycle (∼24 h) and corals had their polyps extended during this incubation period. Water baths were maintained under light and temperature conditions as described above. At the end of the incubation, coral fragments were collected from the chambers and rinsed with filtered seawater to remove diazotrophs present in the mucus that would interfere with the amount of DDN assimilated into the corals. Samples were stored frozen until ^15^N analysis of the animal tissue, Symbiodiniaceae and skeleton fraction. The incubation water was filtered onto pre-combusted (400°C, 4–5 h) GF/F filters and filters were dried in an oven (60°C, 24 h) for ^15^N analysis to quantify DDN of the particles suspended in seawater.

### Sample Treatment Before Analysis

For all coral samples (used for DDN assimilation and natural stable isotope signature analysis), the tissue was removed from the skeleton using an airbrush and homogenized with a Potter-Elvehjem tissue grinder. Homogenates were then separated in several centrifugation steps into the animal and algal fractions according to Grover et al. (2002) and each fraction was subsequently freeze-dried. The remaining skeleton was dried in an oven (60°C, 48 h). To analyze DDN assimilation within the skeletal matrix, a small skeletal piece (0.5 cm) was cut and ground to powder using a CryoMill (Retsch GmbH, Germany). Prior to grinding, the surface area (cm^2^) of the small skeletal piece was determined using a caliper. The surface area of the remaining skeleton was determined using the single wax-dipping technique (Veal et al., 2010).

### DDN Assimilation Analysis

For each sample (host tissue, Symbiodiniaceae, skeleton, seawater particles), the particulate organic carbon (PC) and nitrogen (PN) content (in ng) together with its ^15^N content in atom% were quantified using a Delta V Plus isotope ratio mass spectrometer (Thermofisher Scientific, Germany) coupled via a ConFlo III interface to a FlashEA 1112 HT elemental analyzer (Thermofisher Scientific, Germany). The coefficients of variation are 0.32% for PC and 0.04% for PN content, and the standard deviation in ^15^N atom% measurements is within 0.0001%.

Diazotrophically-derived nitrogen assimilation rates in the different compartments were calculated as follows according to Montoya et al. (1996):

Ne⁢x⁢c⁢e⁢s⁢s15=15Ns⁢a⁢m⁢p⁢l⁢e-15Nc⁢o⁢n⁢t⁢r⁢o⁢l

$$DDNassimilation\;=\left[atom\%{(}^{15}N_{e\,x\,c\,e\,s\,s})/\left(t\times atom{\%}^{15}N_{i\,n\,c\,u\,b\,a\,t\,i\,o\,n\,w\,a\,t\,e\,r}\right)\right]\times \left[\mu \,g\,P\,N_{s\,a\,m\,p\,l\,e}/S\,A\right]$$

*^15^N_*sample*_* is the ^15^N content of the samples after exposure to ^15^N_2_ enriched seawater and *^15^N_*control*_* is the natural ^15^N content of control samples without ^15^N_2_ exposure. The enrichment of samples (*^15^N_*excess*_*) was considered significant when the value was at least three standard deviations away from the mean obtained on control samples. *^15^N_*incubation water*_* is the theoretical ^15^N of the ^15^N_2_ enriched incubation water at the beginning of the incubation. Based on the volume of the incubation bottle and the quantity of ^15^N_2_ enriched seawater added, this resulted in a theoretical enrichment of ∼9.8 atom %^15^N in the incubation medium of all bottles similar to previous ^15^N_2_ studies on corals (Benavides et al., 2016; Camps et al., 2016). *PN*_*sample*_ is the nitrogen content of the samples and SA and t represent the skeletal surface area of the coral fragments (cm^2^) and the incubation time (h), respectively.

The measured carbon and nitrogen content of each sample was also used to calculate the respective carbon and nitrogen content per skeletal surface area as well as the carbon:nitrogen (C:N) ratio of both host tissue and Symbiodiniaceae.

### Natural Stable Isotope Signature Analysis

Analysis of the natural δ^13^C and δ^15^N stable isotope signatures was performed on the dedicated freeze-dried coral samples (host tissue and Symbiodiniaceae), which had been treated with 10% HCl to remove the inorganic carbon. Samples were analyzed with a Dual pumped SerConH 20–20 isotope ratio mass spectrometer coupled to a Thermo EA1110 elemental analyzer. International reference materials (IAEA-600 and IAEA-CH6, International Atomic Energy Agency) were used for scale calibration of the results to Vienna PeeDee Belemnite (VPDB) for δ^13^C and air for δ^15^N measurements. Precision as determined by repeated analysis of the reference materials and quality controls was better than ±0.20 and ±0.15% for measured δ^15^N and δ^13^C values, respectively. Data are expressed in the standard δ unit notation:

$$\delta X=\left[\left(Rsample/Rreference\right)-1\right]*10{}^{3}$$

where *R* = ^13^C/^12^C for carbon and ^15^N/^14^N for nitrogen, and reported relative to VPDB for carbon and to air N_2_ for nitrogen.

### Statistical Analysis of Physiological Data

Generalized Linear Models (GLM) were used to assess whether there were any differences in the response variables DDN assimilation rate, nitrogen content, C:N ratio, natural δ^15^N and δ^13^C given the factors Season, Depth and/or Compartment (i.e., host tissue, Symbiodiniaceae, skeleton). The boxcox transformation procedure as implemented in the R-package *MASS* (Venables and Ripley, 2002) was applied to all the response variables, except natural δ^15^N and δ^13^C, to ensure a normal distribution of the residuals (as confirmed using normal distribution histogram plots and the Shapiro-Wilk test) and equal variances among groups (as confirmed using the Levene’s test [R-package *car* (Fox and Weisberg, 2019)]. For each response variable, the best model fitting the data was selected based on the corrected Akaiki Information Criterion (AICc) using the R-package *AICcmodavg* (Mazerolle, 2020). Outliers were identified based on the fitted model using the outlierTest function of the R-package *car* (Fox and Weisberg, 2019). One Symbiodiniaceae sample (winter from 25 m) was identified as an outlier and removed from the natural stable isotope ^15^N signature dataset, and all steps described above were repeated to ensure the best possible GLM fit. In all cases, the full model including all interaction terms (response variable ∼ Season × Depth × Compartment) was selected. Main and interactive effects were tested for with a Type III Sum of Squares ANOVA on the fitted GLM using the Anova() function from the package *car* (Fox and Weisberg, 2019). In the case of the C:N ratio, a heteroscedasticity-corrected coefficient covariance matrix (HCCM) was used as the Levene’s test indicated unequal variances among groups. The R-package *emmeans* (Lenth et al., 2020) was used to calculate the estimated marginal means given the fitted models and compute the biologically relevant pairwise contrasts. The General Linear Hypothesis Testing [glht function implemented in the R-package *multcomp* (Hothorn et al., 2008)] was used to obtain more exacting adjustments for the multiple comparisons. Boxplots were generated using the R-package *ggplot2* (Wickham, 2016).

To assess the trophic strategy of the corals across the depth gradient, biplots of natural δ^13^C and δ^15^N stable isotope signatures were generated using the R-package *SIBER* (Jackson et al., 2011). Ellipses were plotted for the δ^13^C and δ^15^N values of each group (host tissue or Symbiodiniaceae for each season and depth) on the biplot to depict their isotopic space and the amount of ellipse overlap between host and Symbiodiniaceae was assessed (Conti-Jerpe et al., 2020). As proposed by Conti-Jerpe et al. (2020), an ellipse overlap of ≥70% indicates autotrophy, ≤10% indicates heterotrophy, and an overlap of >10 and <70% indicates mixotrophy. To determine whether the trophic niche of the host and Symbiodiniaceae were different at each depth and season, a Euclidean distance matrix was generated for the full dataset and a permutational multivariate analysis of variance (perMANOVA) was then performed under Type III partial Sums of Squares and 9999 permutations under the reduced model. PERMDISP was used to verify homogeneity of multivariate dispersions (*P* = 0.62) (Anderson, 2006). Significant differences between the centroids of groups (*P* < 0.05) would indicate that host and Symbiodiniaceae occupy a different isotopic space.

All above-described analyses were performed in the R environment for statistical computing and graphics version 3.5.0 (R Development Core Team, 2017), except for the perMANOVA, which was conducted using PRIMER6 + PERMANOVA (Clarke and Gorley, 2006; Anderson and Walsh, 2013). Outcomes of all statistical analyses can be found in Supplementary File 1.

### DNA Extraction and Sequencing Library Preparation

From each coral fragment, a small piece (ca. 0.5 cm) was used to extract the genomic DNA from the coral tissue using the Genomic DNA Buffer Set and Genomic-tip 20/G columns (QIAGEN, Germany) and following the manufacturer’s “sample preparation and lysis protocol for tissues.”

Extracted DNA was shipped to Macrogen (Seoul, South Korea) for amplicon sequencing library construction using (I) the 341F (50- TCGTCGGCAGCGTCAGATGTGTAAAGAG ACAG CCTACGGGNGGCWGCAG-30) and 805R (50- GTCTCGT GGGCTCGGAGATGTGTATAAGAGACAG GACTACHVGGG TATCTAATCC-30) primer set that targets the V3-V4 regions of the 16S rRNA gene (Klindworth et al., 2013) and (II) primers (mnifHF 50- TCGTCGGCAGCGTCAGATGTGTATAAGAGA CAG TGYGAYCCNAARGCNGA-30, mnifHR 50- GTCTCGTG GGCTCGGAGATGTGTATAAGAGACAG ADNGCCATCATY TCNCC-30) (Zehr and McReynolds, 1989) targeting the variable region (360 bp) of the dinitrogenase reductase (nifH) gene to specifically identify the diazotrophic community. Illumina adapter sequences were incorporated in the forward and reverse primers (underlined). Amplicons were generated in a 25-cycle PCR [initialization: 95°C for 3 min; amplification of 25 cycles: 95°C for 30 s, 55°C (or 57°C for *nifH*) for 30 s and 72°C for 30 s; final elongation: 72°C for 5 min] using the KAPA HiFi Hotstart ReadyMix (Kapa Biosystems, United States). Amplicons were purified using the Agencourt AMPure XP beads (Beckman Coulter), and followed by a PCR to attach dual indices using the Nextera XT Index Kit v2 (Illumina, United States). Resulting amplicon libraries were purified with the Agencourt AMPure XP beads (Beckman Coulter), quantified on a Agilent 2100 Bioanalyzer (Agilent Technologies, Santa Clara, CA, United States), normalized to 4 nM and pooled in equal proportions. Pooled amplicon libraries were denatured and loaded on the Illumina MiSeq system for paired-end (2 × 300 bp) sequencing.

### 16S rRNA Gene Amplicon Sequence Analyses and Data Processing

The 16S rRNA gene amplicon data was processed using the UNOISE2 pipeline (Edgar, 2016) as implemented in the USEARCH package (version 9.2^2^) (Edgar, 2010). The raw forward (R1) and reverse (R2) sequence. fastq files of the 36 samples contained a total of 7,615,510 reads (ranging between 79,039 and 215,150 reads per sample). R1 and R2 paired reads were merged using -fastq_mergepairs. Primer sequences were trimmed using -fastx_truncate and reads were quality filtered with the -fastq_filter script, generating a filtered fasta file containing 3,133,323 reads with an average length of 424 bp. Unique sequences were identified using the –fastx_uniques script followed by denoising of the sequence dataset with the UNOISE2 algorithm, obtaining 17,937 denoised sequences or “zero-radius OTUs” (zOTU). The usearch_global script was then used to generate an OTU (Operational Taxonomic Unit) table at the 97% similarity level, containing 11,644 OTUs and an average 84,693 reads per sample (range 62,559 and 169,973 reads). The taxonomy was assigned to each OTU based on the SILVA database (release v123) (Quast et al., 2013) using the -sintax algorithm. The OTU table was converted to the HDF5 biom format and taxonomic assignment metadata was added. Unassigned OTUs, and OTUs classified as chloroplast or mitochondria were excluded from the dataset. The final OTU table contained 2,871,134 reads belonging to 11,045 OTUs, with an average of 79,754 reads per sample (min 45,885; max 169,702). The unfiltered OTU table, sample metadata and representative sequences of each OTU are provided in the Supplementary Files 2–4. Raw sequences were deposited in the NCBI Sequence Read Archive (SRA) under accession number PRJNA660928.

The OTU table was rarefied to 45,885 reads per sample, containing 8,794 OTUs. Using PRIMER 6 and PERMANOVA+ (PRIMER-E Ltd., Auckland, New Zealand) (Clarke and Gorley, 2006; Anderson and Walsh, 2013), permutational Multivariate Analysis of Variance (perMANOVA) performed under Type III partial sums of squares and 9999 permutations under a reduced model was used on square root-transformed Bray-Curtis dissimilarity matrices to statistically assess differences in bacterial community diversity between seasons and depths, and a distance-based Redundancy Analysis (dbRDA) ordination was used to visualize these differences. PERMDISP was used to test for the assumption of homogeneity of multivariate dispersions prior to perMANOVA (F_5,30_ = 1.7679, *p* = 0.3342). The phyloseq package (McMurdie and Holmes, 2013) integrated in R was used to generate relative abundance plots of the overall microbial communities.

### Bacterial *nifH* Gene Amplicon Sequence Analyses and Data Processing

The bacterial *nifH* gene amplicon dataset was processed using the TaxADiva (TAXonomy Assignment and DIVersity Assessment) pipeline (Gaby et al., 2018), followed by Minimum Entropy Decomposition [MED; (Eren et al., 2015)]. In short, raw forward and reverse reads (total of 5,475,486 reads with length of 301 bp; min 102,276 reads; max 169,769 reads) were merged with Paired-End read mergeR [PEAR; (Zhang et al., 2014)] and merged reads <300 and >450 bp were removed. Primer sequences were removed using Prinseq (both sides 17 bp)^3^ and chimeric sequences were removed with VSEARCH (Rognes et al., 2016). MED was used to cluster the remaining 802,201 merged reads into unique oligotypes and identify matches to the *nifH* sequence database [provided with TaxADiva; (Gaby and Buckley, 2014)]. Of the 544 oligotypes identified, 94 showed a BLAST hit with the *nifH* sequence database and were selected for further analysis. The remaining oligotypes were queried against the non-redundant database using BLASTx, and 47 oligotypes were identified as potential *nifH*-encoding sequences although their taxonomy remained unclear. The selected 141 *nifH* oligotypes (average length of 324 or 327 bp; representing 167,246 reads; reads per sample: minimum 0 and maximum 36,458 reads) were clustered at the 95% similarity level using USEARCH (Edgar, 2010), resulting in 97 *nifH* OTUs, and an OTU table was generated. The OTU table, metadata and representative sequences can be found in Supplementary Files 5–8.

Representative sequences of each *nifH* OTU were loaded into MEGA 6 (Tamura et al., 2013) and translated to peptide sequences with a length of 108 amino acids for further taxonomic analysis. Peptide sequences were clustered at 97% (i.e., max. 1 amino acid difference), and the representative sequences of the resulting 34 clusters were queried against the non-redundant protein sequence database using BLASTp (with uncultured and environmental sample sequences excluded). Curated RefSeq matches along with the *nifH* sequences previously found in corals (Olson et al., 2009; Lema et al., 2012; Lesser et al., 2018) and several *nifH* paralogs (belonging to Cluster II and IV) were aligned with the 34 novel *nifH* sequences using the ClustalW algorithm. A 113 amino acid long (including gaps) alignment matrix was selected and a Maximum-Likelihood phylogenetic tree reconstruction was performed based on the Whelan and Goldman (WAG) model using 1000 replicate bootstraps to ascertain node support. Final taxonomy of the identified *nifH* sequences was inferred based on their placement within the phylogenetic tree, the taxonomy assigned by the TaxADiva pipeline and the best BLASTp-match. All raw *nifH* sequence data are accessible at the NCBI Sequence Read Archive under accession number PRJNA660928.

### Predictive Functional Profiling of the Microbiome

The PICRUSt2 (Phylogenetic Investigation of Communities by Reconstruction of Unobserved States) pipeline (Langille et al., 2013; Douglas et al., 2020) was used to identify OTUs putatively involved in the nitrogen cycle. Based on the Kyoto Encyclopedia of Genes and Genomes (KEGG; genome.jp/kegg/) (Kanehisa and Goto, 2000), the relevant KEGG orthologs were selected. OTUs that had a Nearest Sequenced Taxon Index (NSTI) value of <0.3 and that were predicted by PICRUSt2 to contain at least one KEGG ortholog involved in each step of a nutrient cycling process (Nitrogen Cycle: nitrogen fixation, nitrification/ammonium oxidation, nitrate reduction/ammonification, and/or denitrification) were selected. The relatively low NSTI cut-off level of <0.3 was chosen to ensure the functional inferences were conservative and significantly more reliable than under default conditions (NSTI < 2). All taxa involved in a given step of the nitrogen cycle together were considered a functional group. The nitrite-to-nitrate step in the nitrification process is, however, only performed by highly specialized microbes possessing the *nxrAB* genes; however, these genes belong to KEGG orthologs K00370 and K00371, which also contain the common *narGZHY* genes involved in denitrification and dissimilatory nitrate reduction to ammonium. Therefore, we ensured that only taxa known to be capable of performing this step (e.g., *Nitrospina*, *Nitrospira*) were included. Data on the relative abundances of functional groups did not follow a normal distribution. As the interaction between season and depth was of interest, we used an aligned rank transformation of the data as implemented in the R-package ARTool (Kay and Wobbrock, 2015) followed by a two-way Type III Analysis of Variance (ANOVA). Subsequently, pair-wise contrasts were analyzed using Estimated Marginal Means with the *emmeans* package (Lenth et al., 2020).

## Results

### DDN Assimilation

**FIGURE 1:**
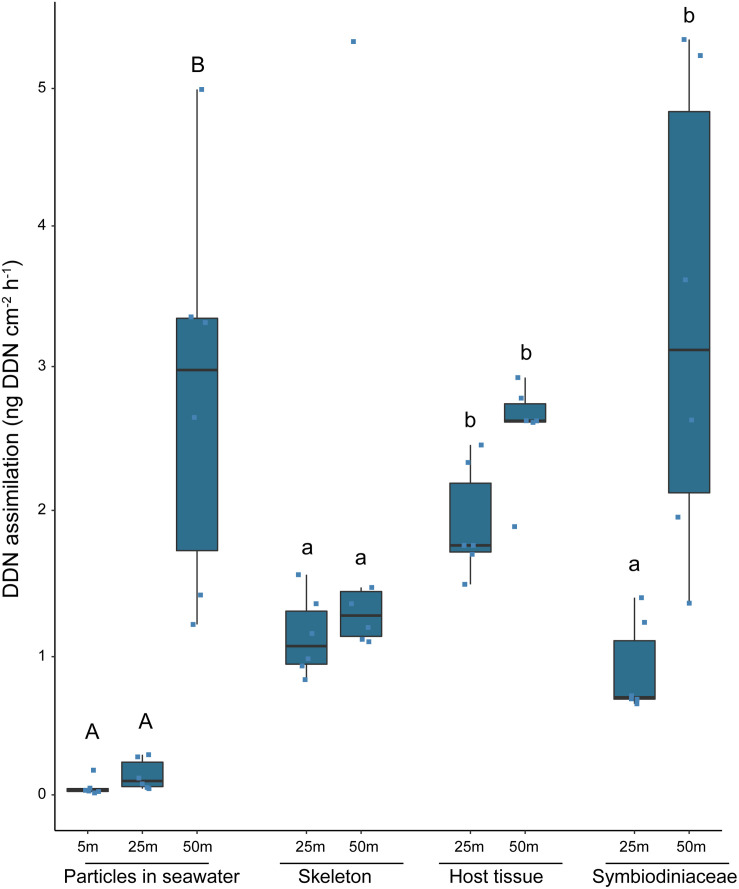
DDN assimilation rates in particles of the incubation water and in the different compartments of *Stylophora pistillata* corals from three different water depths in winter. Values from the three coral compartments at 5 m in winter and values from summer are not shown as no DDN assimilation was detected there. Different lettering above the bars indicates significant differences between the particles in seawater (A, B) and separately between the three coral compartments (a, b) (Generalized Linear Models; significance level: *p* < 0.05).

Diazotrophically-derived nitrogen assimilation by *S. pistillata* in the Gulf of Aqaba was only detected in winter (Figure 1; Supplementary File 1A) but was not detected in samples from 5 m depth. A significant interactive effect was observed between depth (25 m, 50 m) and compartment (skeleton, host tissues, and Symbiodiniaceae) (*P* < 0.001). Specifically, DDN was incorporated at significantly lower levels in the skeleton than the host tissue (*P* = 0.02) and Symbiodiniaceae (*P* = 0.01) in corals from 50 m, but the assimilation rates were similar between the tissue and the Symbiodiniaceae compartments (*P* = 0.73). In contrast, corals from 25 m had higher levels of DDN incorporation in host tissues compared with the Symbiodiniaceae (*P* < 0.001) and skeleton (*P* = 0.01). When assessing the depth effect on each compartment separately, only Symbiodiniaceae showed significantly higher DDN assimilation rates at 50 m than at 25 m (*P* < 0.001). In winter, DDN was also detected in particles in the seawater surrounding the corals. There, significantly more DDN was present in the incubation seawater with corals from 50 m depth than with corals from 5 or 25 m depth (*P* < 0.001), but no difference was detected between the two shallower populations (*P* = 0.064).

### Nitrogen Content and C:N Ratio of *Stylophora pistillata*

**FIGURE 2:**
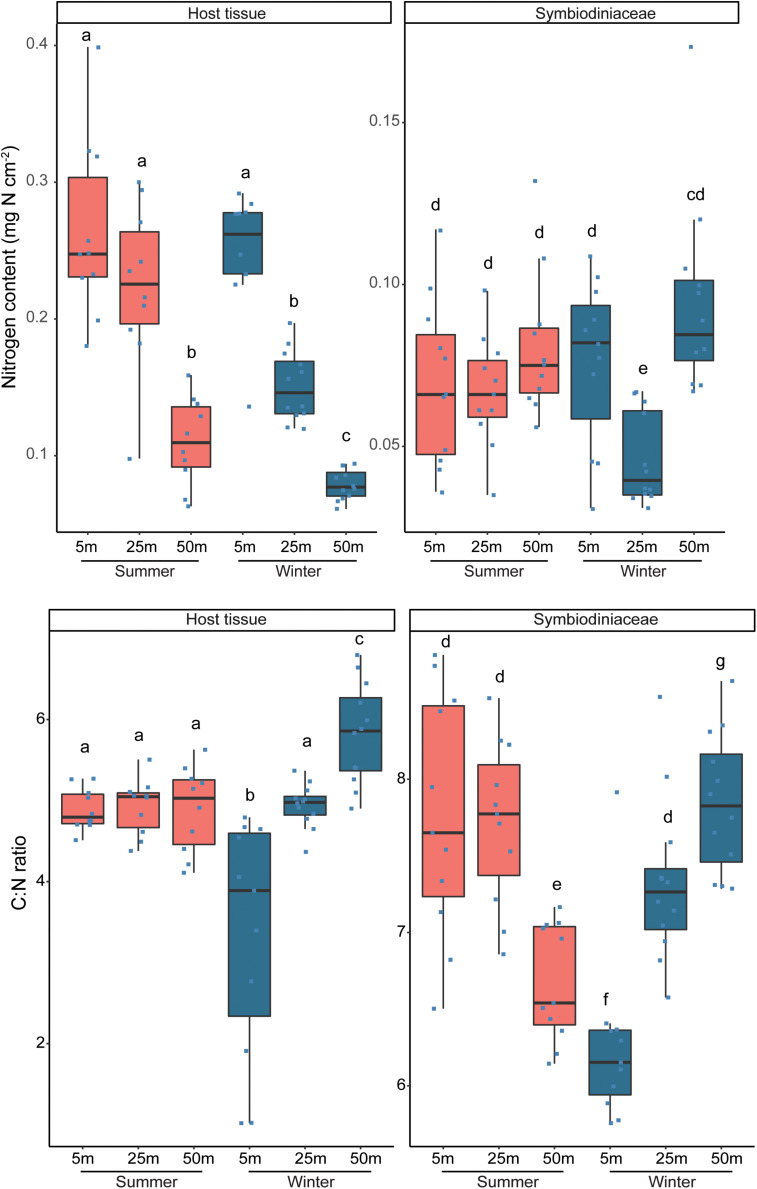
Nitrogen content and atomic C:N ratios of host tissue and Symbiodiniaceae from *Stylophora pistillata* corals collected along a depth gradient (5, 25, and 50 m) in summer and winter. Different lettering (a–g) above the bars indicates significant differences between samples (Generalized Linear Models; significance level: *p* < 0.05). Note the different scaling of the y-axes between host tissue and Symbiodiniaceae.

The nitrogen content per surface area was not affected by season, but a significant interactive effect was observed between water depth and compartment (*P* < 0.001; Figure 2, Supplementary File 1B). Overall, the nitrogen content was significantly higher (*P* < 0.05) in the host tissue than in the Symbiodiniaceae (except at 50 m depth in winter), and while the content in the host tissue significantly decreased with increasing depth, it remained more stable in the Symbiodiniaceae over the depth gradient.

A seasonal (*P* < 0.001) as well as an interactive effect between depth and compartment (*P* < 0.001) were observed for the carbon:nitrogen (C:N) ratio (Figure 2, Supplementary File 1C) which was consistently lower in the host tissues than in the Symbiodiniaceae, during both seasons at each depth (*P* < 0.001). In addition, the C:N ratio significantly increased with increasing water depth for both host and Symbiodiniaceae during winter (all comparisons *P* < 0.02). In summer, this pattern was not observed, except that the C:N ratio in the Symbiodiniaceae at 50 m depth was significantly lower than at 5 m (*P* < 0.001) and 25 m (*P* < 0.001) depths. Seasonal differences in the C:N ratios were observed for the host and Symbiodiniaceae but only at 5 and 50 m depth. At 5 m depth, C:N ratios were higher in summer than winter, while the opposite trend occurred at 50 m depth.

### Natural δ^15^N and δ^13^C Isotopic Compositions

**FIGURE 3:**
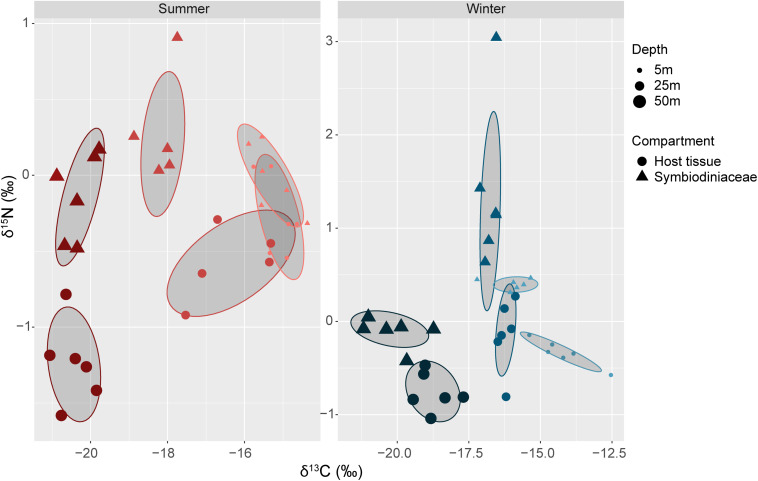
Natural δ^15^N and δ^13^C signature of the coral host and Symbiodiniaceae from *Stylophora pistillata* corals collected along a depth gradient (5, 25, and 50 m) in summer and winter. Note the different scaling of the δ^15^N and δ^13^C axes between summer and winter.

The δ^15^N signatures of corals ranged overall from −1.58 to 0.27 for the host and from −0.48 to 3.04 for the Symbiodiniaceae and a significant interactive effect of the factors season, depth, and compartment was observed (Figure 3; *P* < 0.002; Supplementary File 1D). δ^15^N values were significantly lower in summer than winter (*P* < 0.005) except for the host at 5 m depth and the Symbiodiniaceae at 50 m depth. In addition, the δ^15^N signature also differed along the depth gradient. While the signature in the tissues was significantly lower in corals at 50 m depth compared with those at 5 and 25 m depths during both seasons (*P* < 0.006), the δ^15^N values in the Symbiodiniaceae did not follow a clear pattern related to the depth gradient. Differences in δ^13^C/δ^15^N profiles along the depth gradient were mostly explained by the δ^13^C signature, which significantly decreased along the depth gradient in both host and Symbiodiniaceae during both seasons (all comparisons *P* < 0.04). A clear separation in the δ^13^C/δ^15^N profiles of the host and Symbiodiniaceae was also observed at each depth and season (Figure 3; Supplementary File 1E; perMANOVA *P* < 0.005). Overall, the separation between host and Symbiodiniaceae profiles was clearly reflected in the differences in the δ^15^N signature, whereas the δ^13^C values were similar (±1%). The δ^15^N signature was consistently lower in the host than in the Symbiodiniaceae during both seasons and across water depths (*P* < 0.001), with the exception of corals at 5 m depth in summer showing partial overlap in the host and Symbiodiniaceae isotopic space (perMANOVA *P* = 0.39).

### Bacterial Community and Diazotrophs Associated With *Stylophora pistillata*

**FIGURE 4:**
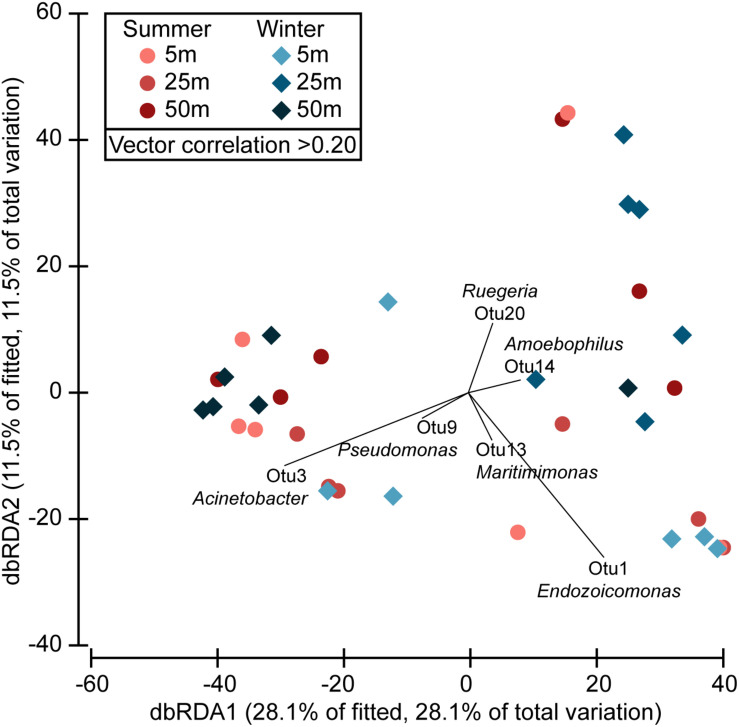
Differences in the diversity of the *Stylophora pistillata* microbiome. Beta diversity of the microbiota of *S. pistillata* collected along a depth gradient (5, 25, and 50 m) in summer and winter is presented in a distance-based redundancy analysis (dbRDA) ordination plot. Correlation vectors indicate the main bacterial groups driving the differences observed.

The prokaryotic community of *S. pistillata* was diverse and relatively variable but showed a separation into two groups: one dominated by Oceanospirillales (genus *Endozoicomonas*) and the other by Pseudomonadales (genera *Acinetobacter* and *Pseudomonas*) (Figure 4, Supplementary Figure 1). Differences in the prokaryotic communities were observed between the samples collected at 50 m depth compared with those collected at 5 and 25 m depth in winter (*P* = 0.029 and *P* = 0.003, respectively), as well as between samples collected at 25 m depth in summer and winter (*P* = 0.019). However, the main driver of the differences observed was the inverse relationship between the abundances of *Endozoicomonas* OTU1 and *Acinetobacter* OTU3, following the two main groups identified (Figure 4). No clear patterns in depth distribution of these two groups were detected (Supplementary Figure 1) suggesting that their abundance might not be controlled by the prevailing environmental conditions but rather by other factors such as the corals metabolic activity (Yang et al., 2017).

The bacterial dinitrogenase reductase (*nifH*) gene was amplified in 5 out of 6 samples of *S. pistillata* collected at 25 m depth in winter and at 50 m depth in summer. No amplification was observed in samples collected at 5 m depth in winter (0/6 samples), and very limited amplification was observed in samples collected at 50 m depth in winter (3/6 samples) and at 5 m (2/6 samples), and 25 m (1/6 samples) depths in summer. Amplicons identified as *nifH* were translated to amino acid sequences, and these operational protein units (OPUs) were used to identify the diazotrophs associated with *S. pistillata*. The majority of the 34 OPUs matched Cyanobacteria (Leptolyngbyaceae, Oscillatoriales, and Pleurocapsales), Alphaproteobacteria (Rhodobacterales and Rhizobiales), Deltaproteobacteria (Desulfobacterales and Desulfovibrionales), Gammaproteobacteria, as well as some Planctomycetes and Verrucomicrobia (Supplementary Figure 2). The taxonomy of three OPUs (MED1952, MED3995, and MED0527) was unclear as it matched to *nifH* amino acid sequences of both Bacteroidetes and Spirochaetes (Supplementary Figure 2). Similarly, the resolution of the Gammaproteobacteria *nifH* OPU sequences was insufficient to determine the taxonomy at lower taxonomic levels. For example, MED2809, MED0173, MED1255, MED4098, and MED5869 matched well to the genera *Pseudomonas*, *Vibrio*, *Klebsiella*, and *Azotobacter*, and MED4035 and MED4102 matched to sequences from the orders Chromatiales and Methylococcales (Supplementary Figure 2). Comparing our *nifH* OPUs with those found in previous studies on coral-associated diazotrophs, the 4 OPUs identified as Rhodobacteraceae (MED3428, MED1445, MED1502, and MED5939) clustered well with sequences previously retrieved from *S. pistillata* from the Great Barrier Reef (Lesser et al., 2018) (Supplementary Figure 2). In addition, *Bradyrhizobium* MED3491, Rhodobacteraceae MED1502 and Planctomycetes MED2810, as well as the OPUs with unclear taxonomy MED1952, MED3995, and MED0527 were closely related to sequences obtained from *Acropora millepora*, *A. muricata*, and *Pocillopora damicornis* (Lema et al., 2012) and *Montipora* spp. (Olson et al., 2009) (Supplementary Figure 2).

**FIGURE 5:**
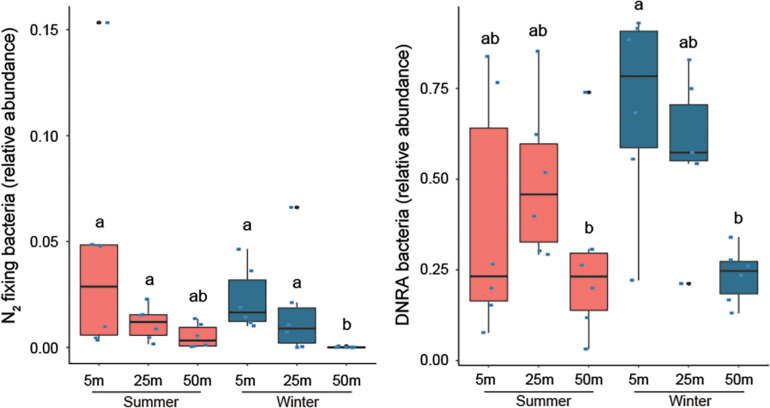
Relative abundance of bacteria putatively capable of N_2_ fixation and dissimilatory nitrate reduction to ammonium (DNRA) associated with *Stylophora pistillata* corals collected along a depth gradient (5, 25, and 50 m) in summer and winter. Different lettering (a, b) above the bars indicates significant differences between samples (Generalized Linear Models; significance level: *p* < 0.05).

PICRUSt2 analysis was used to infer the function of each OTU based on its similarity in the 16S rRNA sequence with microbes whose genome has been sequenced in order to identify bacteria that may be involved in the nitrogen cycle and estimate their abundance. This approach predicted various potential diazotrophs belonging to the Cyanobacteria, Alpha-, Delta-, and Gammaproteobacteria, which was consistent with the *nifH* sequencing results. However, it also predicted multiple Beta- and Epsilonproteobacteria and Clostridiales to be capable of N_2_ fixation, suggesting that our *nifH* amplicon sequencing may have missed certain taxa. Overall, the abundance of potential diazotrophs was low at 1.3% (±1.7%) and highly variable across samples. However, there appeared to be a negative relationship between diazotroph abundance and depth (*P* = 0.013; Figure 5), particularly when comparing corals collected from 5 to 50 m depth (*P* = 0.01). Notably, diazotrophs were nearly absent in corals collected at 50 m in the winter, despite clear N_2_ assimilation measured in these corals. Relatively high levels of bacteria capable of dissimilatory nitrate reduction to ammonium (DNRA) were predicted to be present in *S. pistillata* (Figure 5, Supplementary File 8), including *Endozoicomonas*, *Pseudomonas*, and *Photobacterium*. Depth also had a significant impact on the abundance of these bacteria (*P* = 0.003), with significantly less at 50 m than at 5 m (*P* = 0.01) or 25 m (*P* = 0.006) depths. *Pseudomonas* bacteria were also the main microbes predicted to be capable of denitrification, but the relative abundance of these denitrifying bacteria was not influenced by either season or depth. No microbes involved in nitrification were detected.

## Discussion

### DDN Assimilation by *Stylophora pistillata* Under Varying Environmental Conditions

The quantification of DDN assimilation in *S. pistillata* at three different depths (5, 25, and 50 m depth) and two seasons (summer and winter), points to a significant assimilation only in corals from 25 to 50 m depth during winter. An increasing DDN assimilation with water depth aligns with previous findings in corals from the Gulf of Aqaba (Grover et al., 2014; Bednarz et al., 2017, 2018). This higher DDN assimilation in mesophotic corals during winter may be related to a higher demand for nitrogenous compounds, as the nitrogen content of corals significantly decreased and the C:N ratio significantly increased with increasing water depth. This higher C:N ratio may have stimulated the uptake of DDN in mesophotic corals during winter. The increased DDN assimilation in mesophotic corals may also be related to reduced photosynthesis rates under low light conditions on mesophotic reefs. An inverse relationship between rates of photosynthesis and DDN assimilation has been previously observed in scleractinian corals from the Caribbean and Hawaii, and can result from an inhibition of nitrogenase by hyperoxia under high photosynthesis rates (Lesser et al., 2019). Finally, corals acquire DDN also from heterotrophic feeding on planktonic diazotrophs with assimilation rates that may greatly exceed those of DDN acquired from coral-associated diazotrophs (Table 1; Benavides et al., 2016; Meunier et al., 2019). The occurrence and abundance of planktonic diazotrophs can vary along the depth gradient with highest *nifH* gene copy numbers retrieved from the plankton community at 40–60 m water depth in the Gulf of Aqaba (Foster et al., 2009). If these planktonic diazotrophs are actively fixing N_2_, such depth-related differences in their abundances may have caused the increased DDN assimilation in mesophotic corals as observed here and previously (Bednarz et al., 2017). A coral feeding experiment with ^15^N pre-labeled planktonic diazotrophs collected along a water depth gradient will help to evaluate their role for the nitrogen budget of shallow and mesophotic corals.

In summer, we did not detect any DDN assimilation associated with corals from all three water depths. Low DIN concentrations in the seawater have been suggested to enhance the activity of coral-associated diazotrophs in summer and that this DDN represents an important additional nitrogen source for the coral holobiont during oligotrophic conditions in summer (Cardini et al., 2015). As DIN concentrations in seawater remained low throughout summer, we expected also an active coral-associated diazotrophic community and detectable DDN assimilation rates in the present study. As this was not the case, our results suggest that DDN assimilation by corals is unrelated to seasons and may instead be regulated by temporal changes in environmental conditions and subsequent food availability in seawater. This further reinforces the idea that the availability of DDN to corals is independent of coral-associated N_2_ fixation and is rather derived from predation on planktonic diazotrophs. In the northern Gulf of Aqaba, diazotrophic communities in the upper water column shift from mainly heterotrophic populations in summer to predominantly autotrophic diazotrophs in winter including the cyanobacterium *Trichodesmium* (Rahav et al., 2015). Rahav et al. (2015) reported highest planktonic N_2_ fixation rates during a *Trichodesmium* bloom in winter. Here, we observed ∼ 3-times higher Chl *a* concentrations in coastal waters during winter indicating an increased phytoplankton abundance compared to summer. Also, Foster et al. (2009) measured highest rates of up to 1.9 nmol N l^–1^ d^–1^ in winter/spring when the water column was mixed, while rates during summer ranged from undetectable to 1.2 nmol N l^–1^ d^–1^. DDN assimilation by corals may thus also depend on the community composition, abundance and activity of planktonic diazotrophs in the water column. Overall, the absence of DDN assimilation measured here in summer suggests that corals may indeed rely mostly on planktonic diazotrophs for DDN acquisition and that coral-associated diazotrophs only play a minor role.

### Linking DDN Assimilation With the Coral-Associated Bacterial Community Composition

We also observed a mismatch between DDN assimilation and the presence of coral-associated bacterial diazotrophs. Various Proteobacteria and Cyanobacteria were the most prevalent diazotrophs identified in *S. pistillata*. Some of the *nifH* OPUs identified here clustered well with sequences previously retrieved from *S. pistillata* from other locations such as the Great Barrier Reef (Lesser et al., 2018), but also with sequences obtained from *Acropora millepora*, *A. muricata*, *Pocillopora damicornis* (Lema et al., 2012), *Montipora* spp. (Olson et al., 2009), and the temperate scleractinian coral *Oculina patagonica* (Bednarz et al., 2019). This indicates that some bacterial diazotroph taxa commonly associate with various coral species from different geographical locations. However, *nifH* gene amplification was rather inconsistent among samples and did not follow the same pattern as the DDN assimilation results. For example, corals from 50 m during winter consistently showed the highest DDN assimilation rates among all samples, despite limited *nifH* amplification in those samples (3/6 samples) and very low levels of diazotrophs predicted using functional profiling of the microbiota. Since the presence of coral-associated diazotrophs was highly variable among colonies and across depth and season, our results suggest that diazotrophs form transient associations rather than a true symbiosis with corals. This finding indicates that DDN assimilation may be largely independent from coral-associated bacterial diazotrophs and may instead result from the activity of and predation on planktonic diazotrophs.

Our results indicate that not only diazotrophs, but also bacteria putatively capable of DNRA were predicted to be less abundant in mesophotic corals than in shallow corals. The recycling of nitrogen by converting nitrate to bioavailable ammonium through DNRA prevents a net loss of nitrogen from the holobiont. The lower abundance of bacteria predicted to be involved in DNRA or N_2_ fixation along the depth gradient suggests that there may be a relatively low turnover or even loss of nitrogen from the holobiont at greater depth. More heterotrophic feeding by these corals, may compensate for this nitrogen loss. Although we used a relatively conservative approach (NSTI cut-off < 0.3), it is important to remain cautious when interpreting results of functional inferences based on 16S rRNA gene amplicon sequences as the reliability of the predictions not only depends significantly on how closely related OTUs are to a microbe whose genome has been sequenced (i.e., NSTI), but also because lateral gene transfer among bacteria and mutations may have altered a microbe’s catabolic and anabolic capacities and behavior, compared with its taxonomically close relatives.

Our results further indicate that the presence of bacterial *nifH* genes does not necessarily imply nitrogenase activity since *nifH* amplification was detected in some samples that showed no measurable DDN assimilation rates. A previous study also observed a mismatch with lower DDN assimilation rates in corals where genes involved in N_2_ fixation were predicted (using *PICRUSt2*) to be enriched in comparison with other corals (Lesser et al., 2019). Interestingly, Liang et al. (2020) indicated that corals are not necessarily associated with diazotrophs for the acquisition of newly fixed nitrogen. They found for several coral species from the South China Sea that the nutrient availability in the reef water could completely meet the nitrogen demand of coral holobionts, even without biological N_2_ fixation by coral-associated diazotrophs. Diazotrophs may instead exhibit other biological functions that are beneficial to the host, such as carbon fixation by cyanobacteria. Coral larvae, however, have been shown to associate with planktonic diazotrophs that are actively fixing N_2_ likely as an additional nitrogen source (Lema et al., 2014a, 2016), but whether these associated diazotrophs continue to provide fixed nitrogen to the juvenile and adult coral hosts remains unclear.

As *nifH* levels do not seem to correlate with function, we suggest caution when interpreting *nifH* gene-based studies looking at the diazotrophic community associated with corals and their abundance. Additionally, our results and those of others (Lesser et al., 2018; Bednarz et al., 2019) reported significant non-target amplification that may impact study outcomes. Therefore, we urge the community to optimize and validate molecular techniques for studies into diazotrophs within coral holobionts, and use holistic approaches combining DDN assimilation experiments with molecular analyses.

### Linking DDN Assimilation With the δ^15^N Signature of Corals

One of the major questions related to N_2_ fixation within the coral holobiont is whether DDN contributes significantly to the nitrogen requirements of the coral holobiont. Some insights can be gained from the δ^15^N signature of the coral host and Symbiodiniaceae. A δ^15^N value close to 1–2% have traditionally been attributed to a large input of DDN in coral reef organisms (Yamamuro et al., 1995; France et al., 1998; Lesser et al., 2007). Here, the δ^15^N values were mainly negative for the host and close to zero for the Symbiodiniaceae during both seasons, indicating a significant use of ^15^N-depleted nitrogen sources. Such low and even negative δ^15^N values have been previously reported for corals in the Red Sea (Alamaru et al., 2009), while corals from other regions have generally positive δ^15^N values (Wilkinson and Sammarco, 1983; Muscatine et al., 2005; Swart et al., 2005; Nahon et al., 2013). The seasonal comparison of δ^15^N values indicates more negative values during summer when no DDN assimilation by corals was detected. As DDN assimilation does not lead to negative δ^15^N signatures values in an organism’s tissues, our observations suggest that nitrogen sources other than DDN are contributing mainly to the coral’s nitrogen budget. This is in agreement with a previous study showing that nitrogen from N_2_ fixation only plays a minor role in the Gulf of Aqaba, while nitrate-utilizing cyanobacteria form the primary energy source at the base of the food web (Aberle et al., 2010). Nitrate derived from aerosols has been suggested to support possibly all the new production during summer by supplying ca. 35% of the dissolved inorganic nitrogen to the euphotic zone during the stratification period (Chen et al., 2007). Nitrate from aerosols has on average a very low δ^15^N value of −2.6% (Wankel et al., 2010), while nitrate from deep water has on average a δ^15^N of +5% and becomes available to the euphotic zone during water column mixing in winter (Casciotti et al., 2008). Thus, the use of nitrate from aerosols during summer stratification and the use of nitrate derived from both aerosols and deep water in winter by primary producers might explain the lower δ^15^N values of *S. pistillata* in summer. Another reason for the overall low δ^15^N values of *S. pistillata* corals observed here, might result from nitrogen recycling processes by zooplankton and other heterotrophs in the euphotic zone (Checkley and Miller, 1989; Altabet and Small, 1990). Isotopic fractionation during nitrogen recycling can substantially lower the δ^15^N of particulate organic nitrogen especially during nitrate-deplete conditions such as during water column stratification (Checkley and Miller, 1989). As the Gulf of Aqaba is under the influence of water column stratification in summer resulting in reduced DIN availability, a higher amount of recycled nitrogen, with low δ^15^N values, might have reached the corals and decreased their δ^15^N signature in summer compared to winter. Additional work is required to investigate how the different available nitrogen sources influence the coral’s δ^15^N signature in summer and winter.

Finally, we observed a clear separation of δ^13^C/δ^15^N profiles between host and Symbiodiniaceae that was mainly driven by differences in the δ^15^N signature. Overall, the host often showed lower δ^15^N but similar δ^13^C values compared with the Symbiodiniaceae. This result cannot be explained by a higher heterotrophic input in the host tissue, as this would have increased both δ^13^C and δ^15^N signatures, following higher and positive values generally observed for zooplankton (Muscatine et al., 2005). Possible explanations, which need to be further explored can be a different nitrogen metabolism and/or nitrogen sources between host and Symbiodiniaceae, non-limitation of inorganic nitrogen by the host allowing maximal fractionation, and/or a higher phosphorus limitation of the host tissue compared to the Symbiodiniaceae (Clarkson et al., 2005; Fogel et al., 2008). In contrast, the δ^13^C values were similar between both compartments suggesting both were using the same carbon source, mainly photosynthates transferred by the Symbiodiniaceae to the host.

### Conclusion

The aims of this study were to quantify the DDN assimilation rates in the holobionts of corals living under different environmental conditions and simultaneously investigate whether these rates are linked to the coral-associated diazo- trophic bacterial community and/or to the coral’s natural δ^15^N signature. The results show a discrepancy between the three parameters, likely because DDN appears to have been primarily acquired through feeding on planktonic diazotrophs and contributes little to the total nitrogen assimilation by *S. pistillata* from the Northern Red Sea. Overall, this study highlights the need to take into account the three proxies to fully understand the contribution of internal and external diazotrophy to the nitrogen budget of scleractinian corals. Understanding the trophic ecology of scleractinian corals and their potential sources of nitrogen are important to assess the animal’s capacity to withstand environmental stress, and to thrive under different environmental conditions. This study shows that DDN is not a major contribution to the nitrogen budget of *S. pistillata* corals from the Red Sea.

## Data Availability Statement

The datasets generated for this study can be found in the NCBI Sequence Read Archive under accession number PRJNA660928.

## Author Contributions

VB and CF-P designed the study. VB, RG, J-FM, MF, and CF-P collected and processed the samples. JW analyzed the sequencing data and conducted the statistics. VB, JW, and CF-P wrote the manuscript. All authors reviewed, edited, and approved the final manuscript.

## Conflict of Interest

The authors declare that the research was conducted in the absence of any commercial or financial relationships that could be construed as a potential conflict of interest.

## Footnotes

1 http://www.iui-eilat.ac.il/Research/NMPMeteoData.aspx

2 https://www.drive5.com/usearch/

3 http://prinseq.sourceforge.net/

## Supplementary Material

Supplementary Figure 1 Overview of the composition of the overall bacterial community associated with *Stylophora pistillata* corals collected along a depth gradient (5, 25, and 50 m) in summer and winter in the Gulf of Aqaba. The relative abundance of each taxon within the community is indicated in percentages (%).

Supplementary Figure 2 Phylogeny of the *nifH* operational protein units (OPU) recovered from *Stylophora pistillata* corals collected along a depth gradient (5, 25, and 50 m) in summer and winter in the Gulf of Aqaba. Reconstruction of the maximum-likelihood phylogenetic tree was based on the *nifH* sequences using the Whelan and Goldman (WAG) model. The 34 newly identified *nifH* OPUs from *S. pistillata* are denoted as “MED” and their closest relative sequence match (accession number; taxon; match, and *e*-value) is listed. Boot strap support >60% is indicated on applicable nodes.
