# Supplementary figures and images for: Unravelling the Importance of Diazotrophy in Corals – Combined Assessment of Nitrogen Assimilation, Diazotrophic Community and Natural Stable Isotope Signatures
Source: Front Microbiol. 2021 Jun 24;12:631244. doi: 10.3389/fmicb.2021.631244 (PMC8264265; doi:10.3389/fmicb.2021.631244)

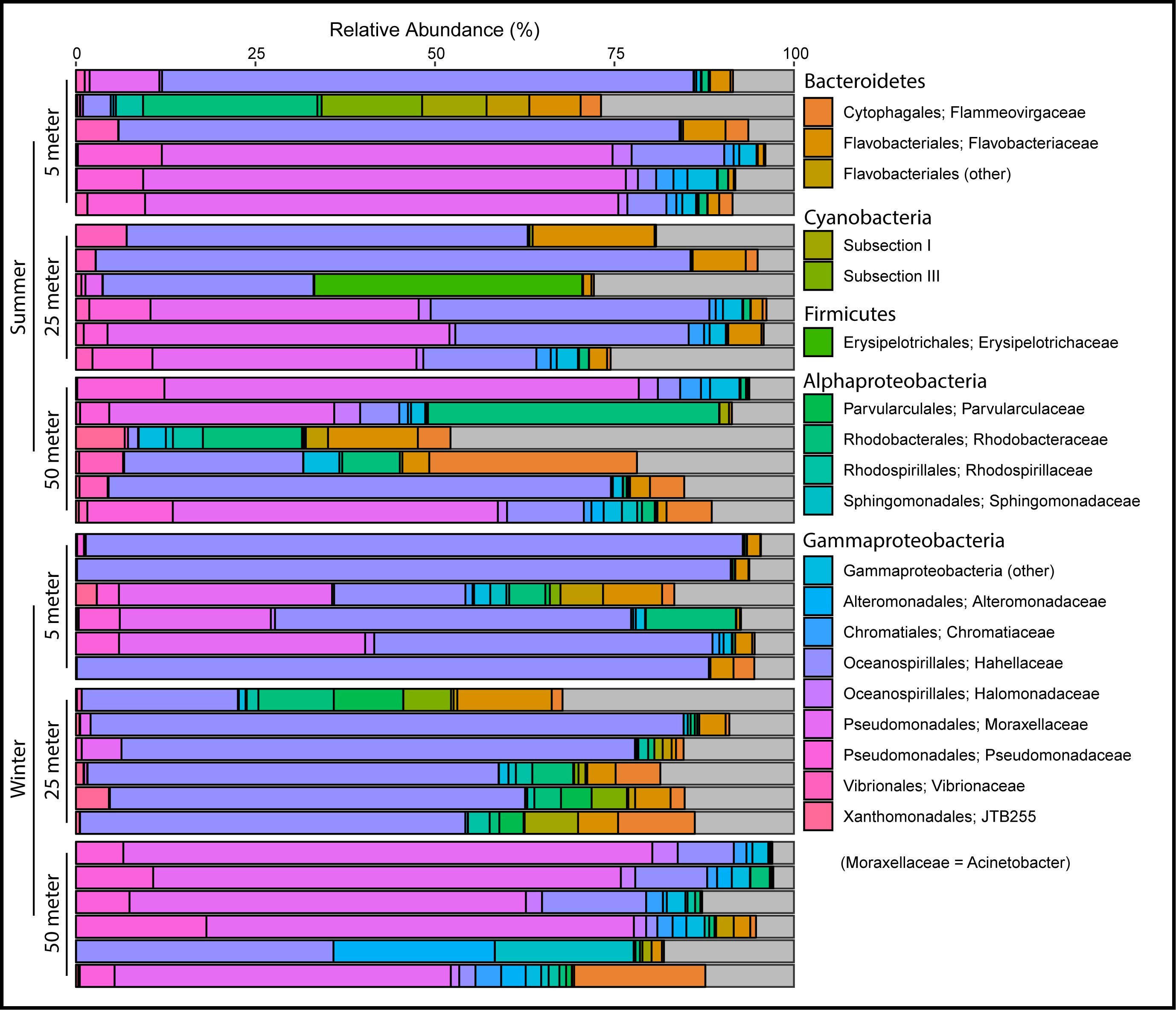

Supplement: Supplementary Figure 1 — Overview of the composition of the overall bacterial community associated with Stylophora pistillata corals collected along a depth gradient (5, 25, and 50 m) in summer and winter in the Gulf of Aqaba. The relative abundance of each taxon within the community is indicated in percentages (%). [file Image_1.TIF]

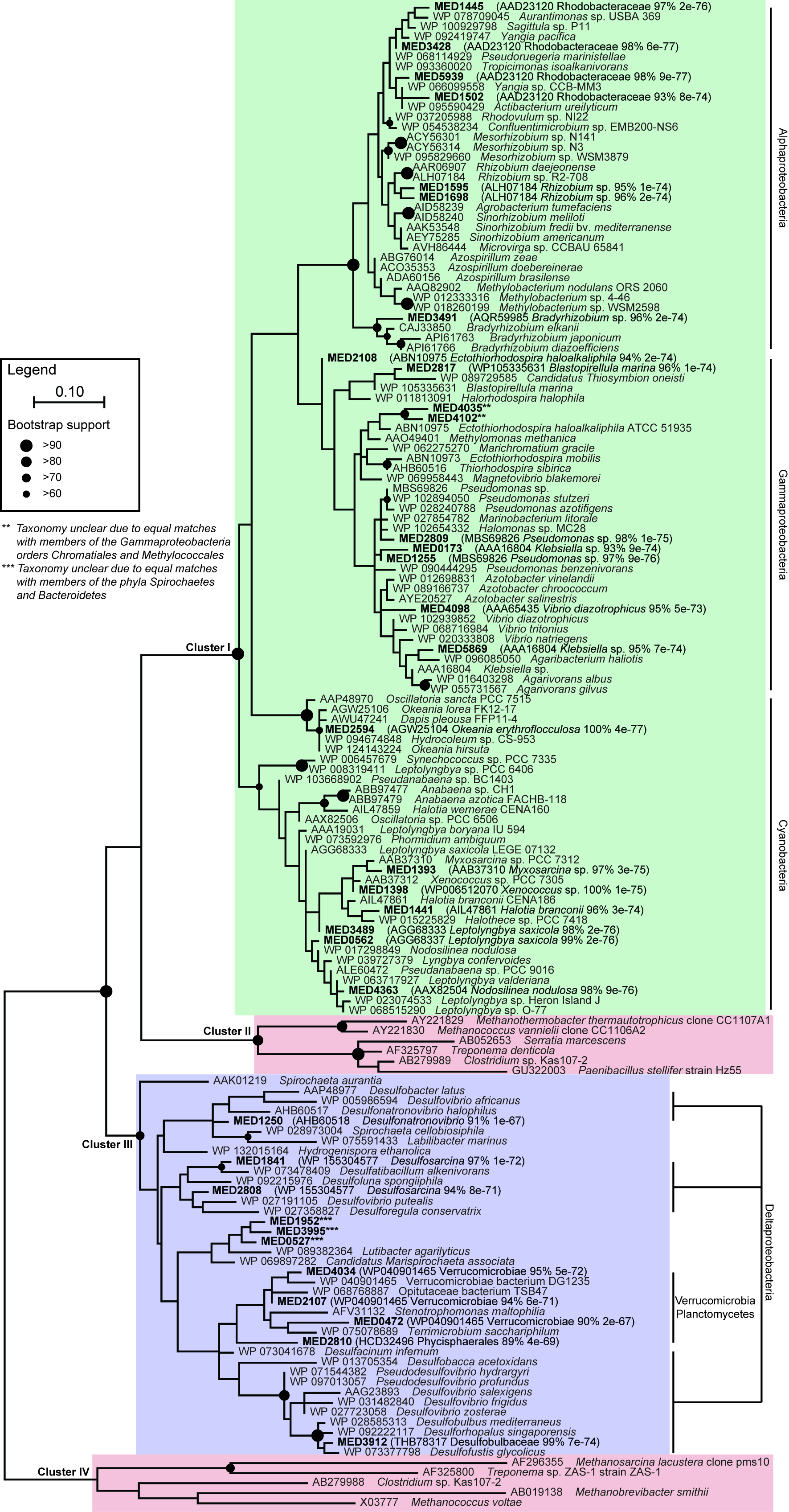

Supplement: Supplementary Figure 2 — Phylogeny of the nifH operational protein units (OPU) recovered from Stylophora pistillata corals collected along a depth gradient (5, 25, and 50 m) in summer and winter in the Gulf of Aqaba. Reconstruction of the maximum-likelihood phylogenetic tree was based on the nifH sequences using the Whelan and Goldman (WAG) model. The 34 newly identified nifH OPUs from S. pistillata are denoted as “MED” and their closest relative sequence match (accession number; taxon; match, and e-value) is listed. Boot strap support >60% is indicated on applicable nodes. [file Image_2.TIF]
